# Thrombophilic risk factors in hemodialysis: Association with early vascular access occlusion and patient survival in long-term follow-up

**DOI:** 10.1371/journal.pone.0222102

**Published:** 2019-09-20

**Authors:** Clemens Grupp, Ilka Troche-Polzien, Johanna Stock, Carsten Bramlage, Gerhard A. Müller, Michael Koziolek

**Affiliations:** Department of Nephrology and Rheumatology, Georg-August University, Goettingen, Germany; University of Missippi Medical Center, UNITED STATES

## Abstract

**Objective:**

Thrombophilic risk factors (TRFs) occur rather frequently in hemodialysis (HD) patients. However, little is known about their significance in HD patients, besides their potential impact on arteriovenous (AV) access failure, with varying results. We examined the effects of a wide variety of TRFs on both early AV fistula occlusion and survival among HD patients in long-term follow-up.

**Methods:**

In this single-center, observational study, 70 consecutive HD patients from our dialysis center were examined with respect to shunt occlusion within the first 2 years after fistula creation and patient survival in a long-term follow-up (at least 16 years). We examined the presence of factor V, prothrombin, and *MTHFR* mutations using real-time fluorescence polymerase chain reaction. Furthermore, antithrombin (AT), protein C, protein S, and antiphospholipid antibodies (APL-Abs) were assessed.

**Results:**

Among the 70 patients, 32 had *MTHFR* mutations, 10 had heterozygous factor V Leiden mutations, 4 had prothrombin mutations, 4 had protein S deficiency, 2 had protein C deficiency, 9 had AT deficiency, and 14 had APL-Abs. 40 patients had shunt occlusion. TRFs were associated with a significantly increased risk for shunt thrombosis (*P*<0.02). Kaplan–Meier analysis with a log-rank test revealed significantly shorter survival in HD patients with TRFs (*P*<0.02). Cox regression analysis showed that the presence of TRFs (*P*<0.05; hazard ratio, 1.94; 95% CI: 1.07–3.56), but not early shunt occlusion, was associated with short patient survival.

**Conclusions:**

TRFs in hemodialysis patients have a strong impact on patient survival and early AV fistula failure; however, patient survival is not significantly affected by early shunt occlusion.

## Introduction

In the general population, thrombophilic risk factors (TRFs) have been reported to be associated with increased mortality, presumably because of venous thromboembolism and associated complications [1–3]. Although TRFs occur even more frequently in hemodialysis patients than in the general population [4–7], little is known about their consequences in this patient group. In particular, information regarding the effects of TRFs on patient survival is lacking. TRFs have been studied in hemodialysis patients, especially with respect to their significance for arteriovenous fistula (AVF) thrombosis [4–6, 8–13]. Failure of an AVF is a serious problem in hemodialysis (HD-) patients, and it represents a major cause of morbidity and hospitalization in this patient group. Therefore, it might have an impact on survival in these patients as discussed by Feldman et al. [14]. The reported 1-year occlusion rates of fistulas vary considerably, and occlusion rates of up to 60% have been reported [15–19]. Coagulation disorders might be of great importance; however, limited and conflicting data exist regarding their relevance [4–6, 8–13]. A better understanding of the relevance of TRFs both for AVF-failure and mortality may help in the development of procedures to reduce these risks.

Therefore, the present study aimed to evaluate the impact of a wide variety of TRFs in HD-patients on the early shunt occlusion rate within 2 years as a short-term effect and, to best of our knowledge for the first time, on patient survival as a long-term effect. The study also assessed whether early AVF occlusion is a significant risk factor for HD-patient survival.

## Subjects and methods

### Study population

The study included 70 outpatients (32 female and 38 male patients) on maintenance hemodialysis treatment at the dialysis unit of the Department of Nephrology in the University Hospital of Göttingen, Germany. The mean patient age was 61 years (range, 32–86 years), and the follow-up period was 16 years (2000–2016). Two patients were lost to follow-up.

Shunt surgery was performed by vascular surgeons at the surgical department in the university clinic. The first AVF was usually a Cimino–Brescia fistula in the forearm (radiocephalic AVF), and if this failed, a fistula was established at the upper arm (brachiocephalic AVF) (Table 1). Patients had neither AVG nor central dialysis catheters. The detailed history of each patient was explored, with particular focus on occlusion of vascular access and other thrombotic events.

**Table 1:** Characterization of patients with and those without thrombophilic risk factors (TRFs) and patients with and without shunt occlusion.

	Patients without TRF (n = 22)	Patients with TRF (n = 48)	*P*-value	Patients without shunt occlusion (n = 30)	Patients with shunt occlusion (n = 40)	*P*-value
Age (years)	61.2±14.4	61.5±12.4	0.92	57.8±13.1	66.5±11.2	<0.005
Sex (male)	13 (59.1%)	25 (52.0%)	0.62	15 (50.0%)	23 (57.5%)	0.39
Diabetes mellitus	3 (13.6%)	14 (29.1%)	0.23	9 (30.0%)	8 (20.0%)	0.33
Coronary artery disease	10 (45.5%)	28 (58.3%)	0.44	17 (56.7%)	21 (52.5%)	0.73
Stroke in history	2 (9.1%)	4 (8.3%)	1.00	1 (3.3%)	5 (12.5%)	0.15
Malignancy in history	4 (18.2%)	6 (12.5%)	0.71	4 (13.3%)	6(15.0%)	0.84
Previous venous thrombotic event	0 (0%)	4 (8.3%)	0.30*	2 (6.7%)	2 (5.0%)	0.77
Fistula location forearm (remaining: upper arm)	17 (77.3%)	37(77.1%)	0.99	24 (80.0%)	30 (75.0%)	0.62

Data are presented as mean ± SD or number (percentage).

Continuous variables (age) were compared using the unpaired *t*-test, and categorical variables were compared using the chi-square test (*Fisher’s exact test). A *P*-value ≤0.05 was considered significant.

The following comorbid conditions were evaluated at the beginning of hemodialysis: cardiovascular disease (stroke or coronary heart disease), malignancy, diabetes mellitus and previous venous thrombotic event (Table 1). During and shortly before the study there were no pregnancies, no patient took oral contraceptives or received estrogen therapy. No patient suffered from nephrotic syndrome. aPTT, INR and platelet count were regularly examined in all patients. No primary abnormalities were observed.

The study was approved by the local ethics committee of Georg-August-University Göttingen and conducted in accordance with the Declaration of Helsinki. Written informed consent was obtained from each participant prior to inclusion into the study.

### Analysis of coagulation factors

#### Prothrombin (20210G>A), factor V Leiden (1691G>A), and MTHFR (677C>T) genotyping

Genotyping was performed with real-time polymerase chain reaction (PCR) involving hybridization probes using Light Cycler (Roche Biochemica, Mannheim, Germany) for prothrombin (20210G>A), factor V Leiden (1691G>A), and *MTHFR* (677C>T) mutations, according to our previously published methods [20, 21].

#### Coagulation assays

Venous blood was collected in 0.1-M buffered trisodium citrate (blood:citrate, 9:1). Two centrifugation steps (10 min at 3000 *g*) were performed in order to obtain platelet-poor plasma. The plasma was aliquoted, frozen, and stored at −20°C until use.

Antithrombin (AT) and protein C activities were determined using the Coamatic LR Antithrombin kit (Chromogenix, Milano, Italy) and the Immunochrom PC kit (Baxter AG, Vienna, Austria), respectively. The functional activity of protein S was determined using the protein S Reagent kit (Dade Behring, Marburg, Germany). The lower limits of the reference ranges for antithrombin, protein C, and protein S activities were 80%, 68%, and 55%, respectively. The anticoagulant response to the addition of activated protein C was detected using the Coatest APC Resistance V kit (Chromogenix, Milano, Italy). The results were expressed as the ratio (APCsr) of the clotting time of plasma with APC to that of plasma without APC. The normal APCsr range was 2.0 to 3.2. The presence of lupus anticoagulant was determined using LA 1 Screening Reagent and LA 2 Confirmation Reagent (Dade Behring). Anti-cardiolipin Abs (aCLs) were determined using an ELISA method (Synelisa Cardiolipin antibodies, Pharmacia & Upjohn, Freiburg, Germany; microplate reader, Tecan, Crailsheim, Germany). The cut-off values for IgG, IgM, and IgA aCLs were 12 GPLU/ml, 6 MPLU/ml, and 10 APL U/ml, respectively.

All functional coagulation assays were performed using an autoanalyzer system (BCS, Dade Behring).

Samples were always taken directly before dialysis.

### Statistical analysis

A preliminary descriptive statistical analysis was performed for all variables. Simple chi-square tests were first used to indicate the univariate dependence between the response variable “thrombophilic risk factor” or “shunt” and the binary explanatory variables. The relationship between the binary response variable “shunt occlusion” and the available explanatory variables was modeled using logistic regression. Logistic regression models the logit transformation of the individual’s event probability “shunt = yes” as a linear function of the explanatory variables. Survival analysis was performed for the variable “time after shunt creation” using the common Cox F-test and log-rank test. The effect of TRFs and other parameters on survival and their potential interactions were examined using Cox regression with backward variable selection. For survival time analysis, patient data were analyzed using Kaplan–Meier estimates and the log-rank test. Patients who were alive and those without existing follow-up data were censored. We have mentioned the 95% confidence intervals (CIs). A *P*-value <0.05 was considered significant. The statistical software Statistica, version 13 (TIBCO Software Inc., Tulsa, OK) was used.

## Results

### Association of thrombophilic risk factors with arteriovenous fistula failure

**Fig 1:**
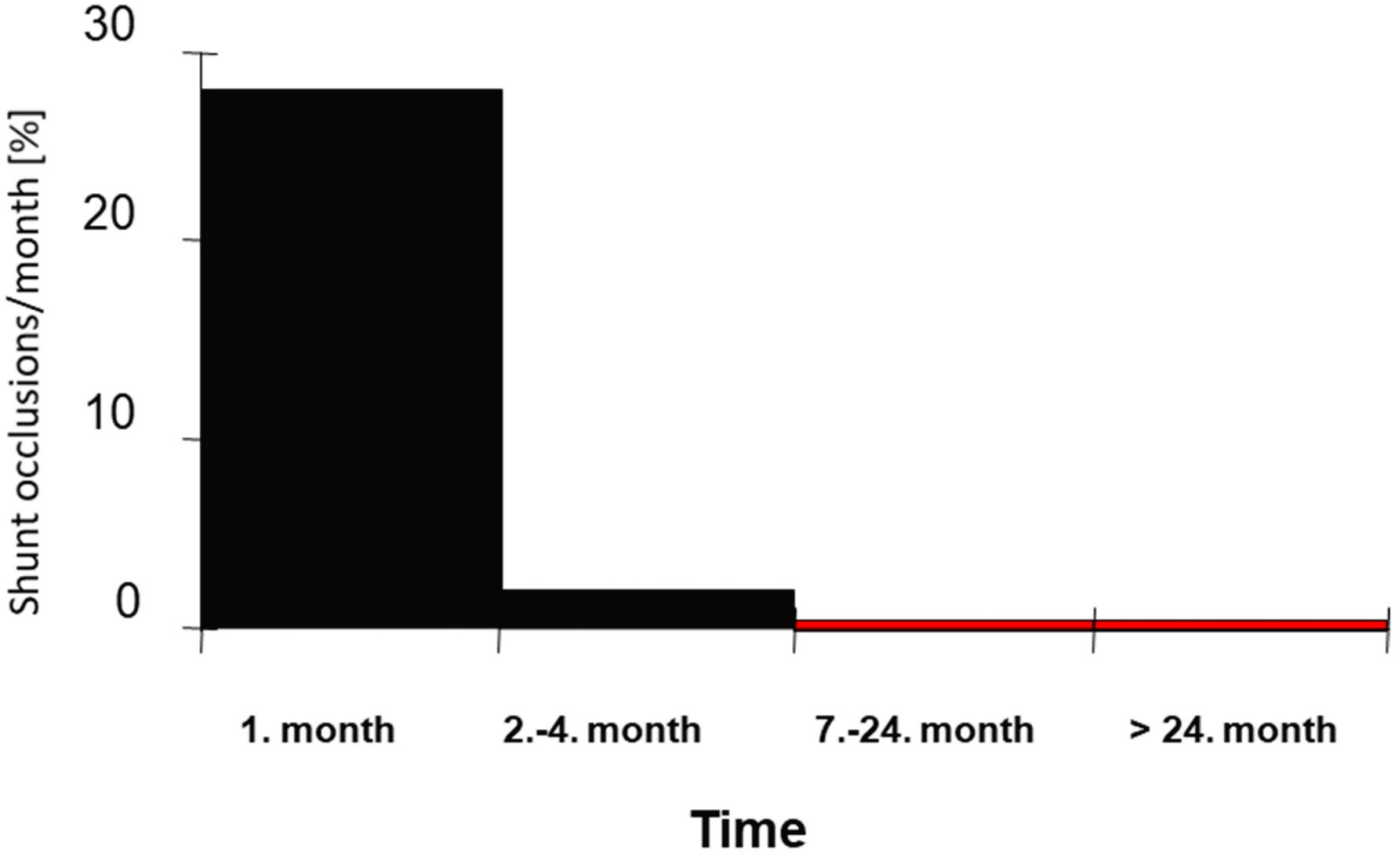
Risk of AVF-occlusion after surgery. Within the first month the occlusion rate was quite high and rapidly decreased during the following months.

Of the 70 HD-patients, 40 (57%) experienced at least 1 AVF occlusion and 24 (35%) had recurrent events within 2 years after the start of dialysis. Most occlusions (28%) occurred during the first month. Within the following 5 months, the occlusion rate decreased to 2% per month, and after 6 months, it decreased to approximately 0.6% per month (Fig 1).

Prior to dialysis 4 patients suffered from thrombosis, 2 of them experienced AVF-occlusion. All patients exhibited MTHFR-mutation, one of these with AVF-failure additionally prothrombin mutation.

**Fig 2:**
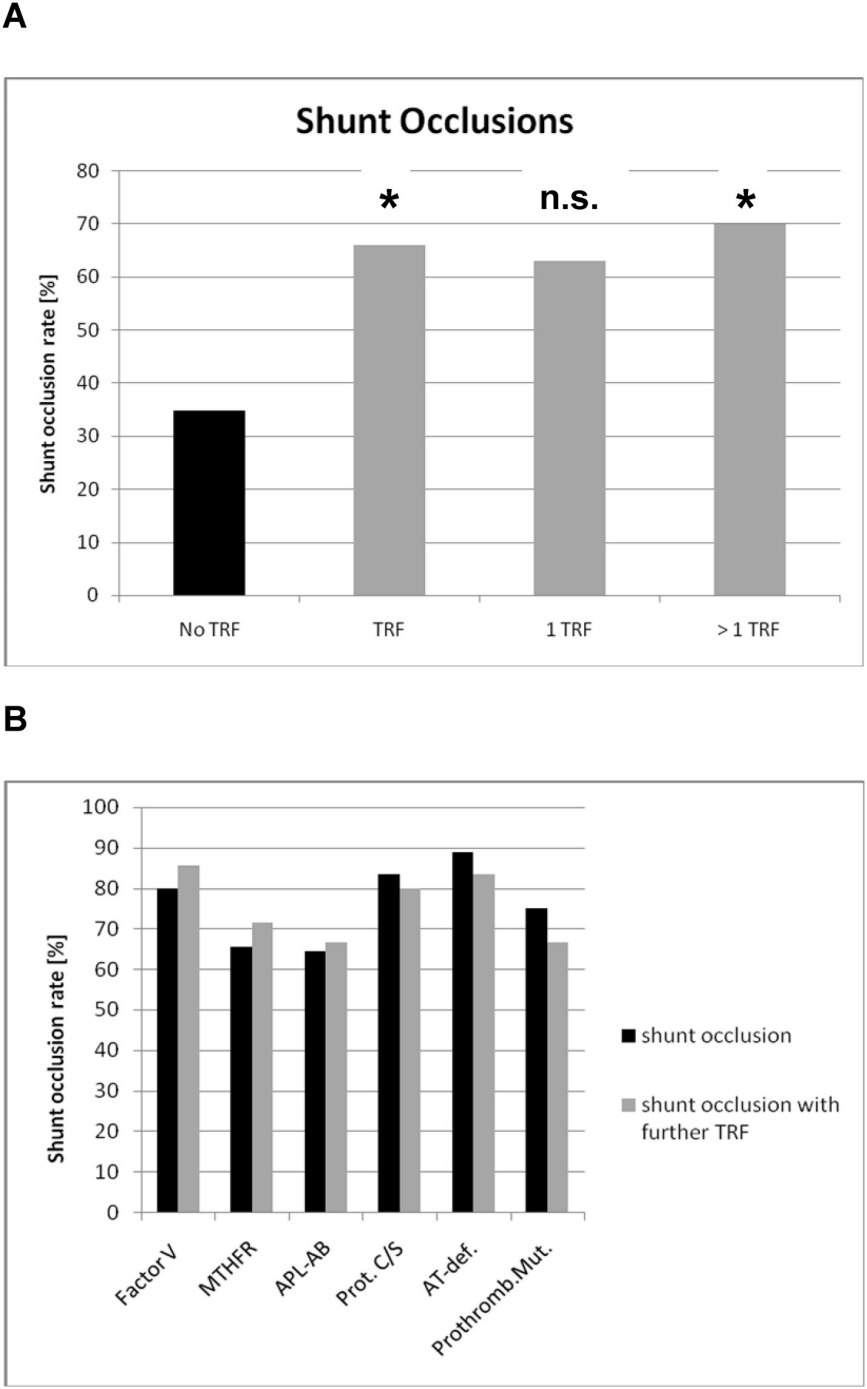
Shunt occlusion rate within 2 years after the start of hemodialysis in patients with thrombophilic risk factors (TRFs) compared to patients without a thrombophilic defect (no TRF). (A) Data of all patients with TRFs (TRF), those with 1 TRF (1 TRF), and those with multiple TRFs (>1 TRF) are shown. *P<0.05 by Fisher’s exact test and chi-square test vs. no TRF; n.s., Fisher’s exact test *P*>0.05. (B) Shunt occlusion rate for the respective TRFs exclusively (dark bars) or combined with at least 1 further TRF (light bars). Factor V, factor V Leiden mutation; MTHFR, *MTHFR* mutation; APL-Abs, antiphospholipid antibodies; Prot. C/S, protein S/protein C deficiency; AT-def., AT deficiency; Prothromb. Mut., 20210G>A mutation of the prothrombin gene.

At least 1 TRF was detected in 68.6% (48/70) of patients, and the frequencies of potential further risk factors for fistula failure are shown in Table 1. Shunt occlusion was noted in 66% of patients with a thrombophilic disorder, and this rate was significantly higher than that in those without a TRF (34.8%; *P*<0.02; odds ratio 3.85; CI: 1.18–12.84; relative risk 1.89; CI: 1.07–3.81; Fig 2A). A single TRF was detected in 40.0% (28/70) of patients, and among these patients, 64.3% (chi-square test *P*<0.05 vs. no TRF) experienced shunt occlusion. Conversely, multiple TRFs were noted in 28.6% (20/70) of patients, and among these patients, 70% experienced shunt occlusion (*P*<0.05 vs. no TRF). The various coagulation disorders are discussed in the following (Fig 2B, Table 2).

**Table 2:** Thrombophilic risk factors (TRFs) examined in this study.

	Prevalence of TRFs in this study population: total number /associated with a further TRF given as number of patients (percentage)	Prevalence in the general population (Europe)	Estimated increased thrombotic risk in the general population[2, 22–24]
Factor V Leiden (G1691A)	**10/7 (14.0/10.0%)**	3–7%	3–5× (heterozygous)
Factor II (prothrombin) (G20120A)	**4/2 (5.7/2.9%)**	1–3%	3–7× (heterozygous)
Antithrombin III deficiency	**9/6 (12.9/8.6%)**	0.03–0.3%*	5–15×
Protein S deficiency	**4/2 (5.7/2.9%)**	0.2–1%	2–3×
Protein C deficiency	**2/2 (2.9/2.9%)**	0.2–0.5%	5–8×
*MTHFR* (C677CT)	**32[2]/14 [0](45.7/20.0%) [homozygous]**	30–40% (heterozygous)	2–3× viahomocystein ⇑
		15% (homozygous)	
Antiphospholipid antibodies	**14/11 (20/15.7%)**		
- Lupus coagulant (LC)	**7/6 (10/8.6%)**	2–3%	10×
- Anti-cardiolipin (AC)	**6/5 (8.6/7.1%)**	1–2%	1.5×
- LC and AC	**1/0 (1.4/0%)**		

Data estimating the prevalence and thrombotic risk are derived from various studies. For details see the text. *hereditary forms

#### Factor V Leiden mutation

Ten (14.0%) patients were identified as heterozygous carriers of this mutation, and all exhibited a lower activated protein C resistance with a mean ratio of 1.62 (range, 1.49–1.80; reference range, 2.00–3.00). Eight patients experienced at least 1 occlusion of vascular access, and of 7 patients with a further TRF, 6 had shunt thrombosis.

#### Protein S/protein C deficiency

In 3 of 4 (5.7%) patients with decreased protein S plasma levels, shunt occlusions were found. Three patients exhibited APC resistance owing to a heterozygous mutation of factor V Leiden. As a factor V mutation may interfere in the assessment of protein S, the diminished levels may be associated with this defect. Both patients with decreased protein C had shunt occlusion.

#### 20210G>A mutation of the prothrombin gene

In 3 of 4 (5.7%) patients with this mutation, shunt thrombosis was noted.

#### Antithrombin deficiency

Nine (12.9%) patients showed AT deficiency. Low AT plasma levels (mean activity, 67%; range, 59–76%) were associated in 8 of 9 patients with shunt thrombosis.

#### Antiphospholipid antibodies

Fourteen (20.0%) patients had antiphospholipid antibodies (APL-Abs; 7 patients with lupus anticoagulant, 6 patients with aCLs, and 1 patient with both APL-Abs; aCL-IgM mean activity, 14.7 MPLU/ml; range, 7.6–34.1 MPLU/ml; aCL-IgG mean activity, 13.8 GPLU/ml; range, 12.7–14.8 GPLU/ml). In 9 of these patients (4 with lupus anticoagulant, 4 with aCLs, and 1 with both APL-Abs), shunt occlusion occurred.

#### MTHFR mutation

In 32 patients with *MTHFR* mutation (30 heterozygous and 2 homozygous), an occlusion rate of 66% (21/32) was observed, and the relative risk increased by a factor of 1.5 (*P*<0.05). In these patients, the plasma homocysteine level increased (without shunt occlusion, 29.9 μml/l; with shunt occlusion, 31.2 μmol/l; *P* = 0.84), while the levels of vitamin B12 and folic acid were within the reference ranges.

### Examination of the influence of other factors on the shunt occlusion rate

Logistic regression analysis showed that the shunt occlusion rate was significantly higher when a TRF was present (*P*<0.01, Table 3). The shunt occlusion rate was also higher in older patients than in younger patients (*P*<0.01). We examined the effect of various factors on shunt occlusion also in univariate dependence by chi-square-test and paired t-test (age). Here we observed a significant effect of TRFs (*P*<0.02) and age (*P*<0.005) on shunt survival as well. No significant associations between the shunt patency rate and sex, diabetes mellitus, cerebral stroke, coronary heart disease, malignancy, previous venous thrombotic event or location of the fistula (forearm/upper arm) could be found (Table 1).

**Table 3:** Risk for early shunt occlusion.

	Odds ratio	Confidence intervals 95% (lower–upper)	*P*-value
Thrombophilic risk factor	6.886	1.63–28.99	<0.01
Age	1.093	1.03–1.16	<0.01
Diabetes mellitus	8.885	0.51–156.07	0.14
Cerebral stroke	0.325	0.08–1.36	0.12
Coronary heart disease	1.728	0.41–7.21	0.45
Malignancy	1.450	0.27–7.77	0.66
Sex	1.179	0.36–3.88	0.79

Logistic regression analysis of the risk for shunt occlusion and various other factors demonstrated a significant association with the presence of thrombophilic risk factors and age.

No significant association was found with sex, diabetes mellitus, cardiovascular disease, or malignancy.

### Thrombophilic risk factors and patient survival

With regard to the examination of survival rates, 2 out of 70 patients were lost to follow-up, as information about their status could not be obtained. 9 patients underwent kidney transplantation. After 16 years of follow-up 7 patients were alive, and of these, 5 had received a renal allograft.

Considering the impact of coagulation disorders on survival, the 2 patients (both with early shunt occlusion) who were alive and still on dialysis at the end of follow-up had no coagulation disorder. Of the 9 transplanted patients, 3 had no TRF, 4 had 1 defect, and the remaining 2 had more than 1 defect. At the end of follow-up, 5 transplanted patients were alive with a kidney graft (3 patients without a TRF and 2 patients with 1 TRF). The 2 surviving patients with 1 TRF (both with shunt occlusion) carried a *MTHFR* mutation (Table 4). A thromboembolic event, as a known cause of death, was observed only in 1 patient without any TRF. The patient died from pulmonary artery embolisms 69 months after the start of dialysis.

**Table 4:** Thrombophilic risk factors and surviving patients.

	Patients overall	Patients alive at the end of the study		
		no TRF	1 TRF	>1 TRF
Patients without a kidney graft	59 (32)	2 (2)	0	0
Patients with a kidney graft	9 (6)	3 (1)	2 (2)	0

Follow-up after 16 years: surviving patients and patients with and without kidney grafts. Of the 70 patients, 2 were lost to follow-up. The 2 surviving transplanted patients with 1 thrombophilic risk factor (TRF) experienced *MTHFR* mutations (homozygous in 1 patient). The number of patients who had a shunt occlusion within the first 2 years of dialysis is presented in brackets.

The effects on survival and the potential interactions of TRFs and early shunt occlusion within 2 years, as well as the parameters of age, sex, coronary heart disease, diabetes mellitus, stroke, and malignancy at the start of dialysis were examined using Cox regression with backward variable selection. Age at the start of dialysis was found to have a strong, significant impact on survival (*P*<0.001; hazard ratio, 1.06; CI: 1.03–1.06). Additionally, the presence of at least 1 TRF was associated with a reduced survival time (*P*<0.05; hazard ratio, 1.94; CI: 1.07–3.56). Besides a borderline significance for malignancy (*P* = 0.06; hazard ratio, 2.4; CI: 0.97–4.27), the other aforementioned parameters, including early shunt failure, did not show any significant effect.

**Fig 3:**
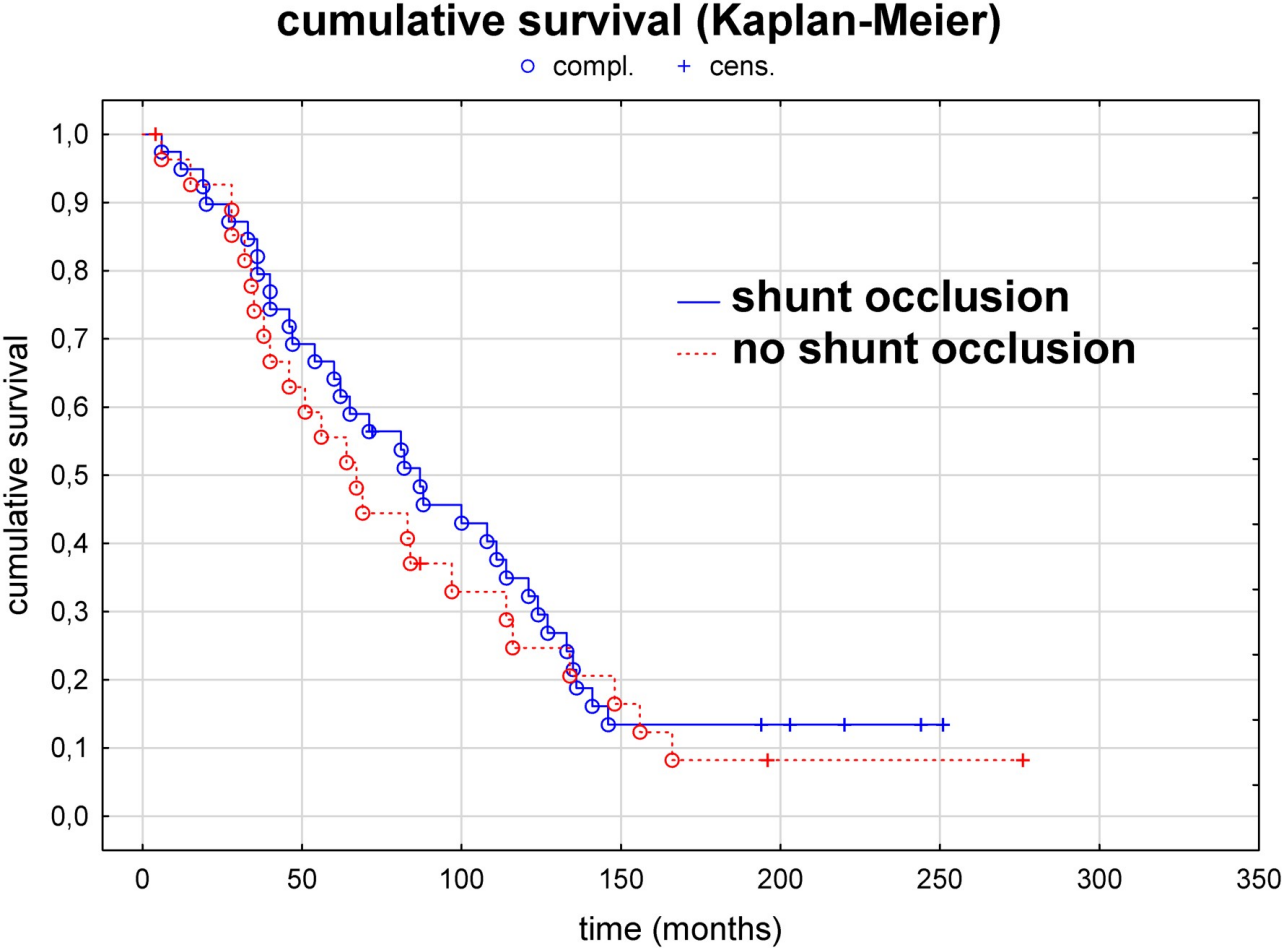
Survival analysis (Kaplan–Meier estimate and log-rank test) of patients with versus those without early shunt occlusion within 2 years. No significant difference is observed between the 2 groups (*P* = 0.63).

In agreement with this finding, on comparing the survival time of the 30 patients without and that of the 40 patients with early shunt occlusion using Kaplan–Meier analysis with a log-rank test, no significant difference in survival time was observed (*P* = 0.54). This finding suggests that early shunt occlusion has no relevant effect on patient survival (Fig 3).

**Fig 4:**
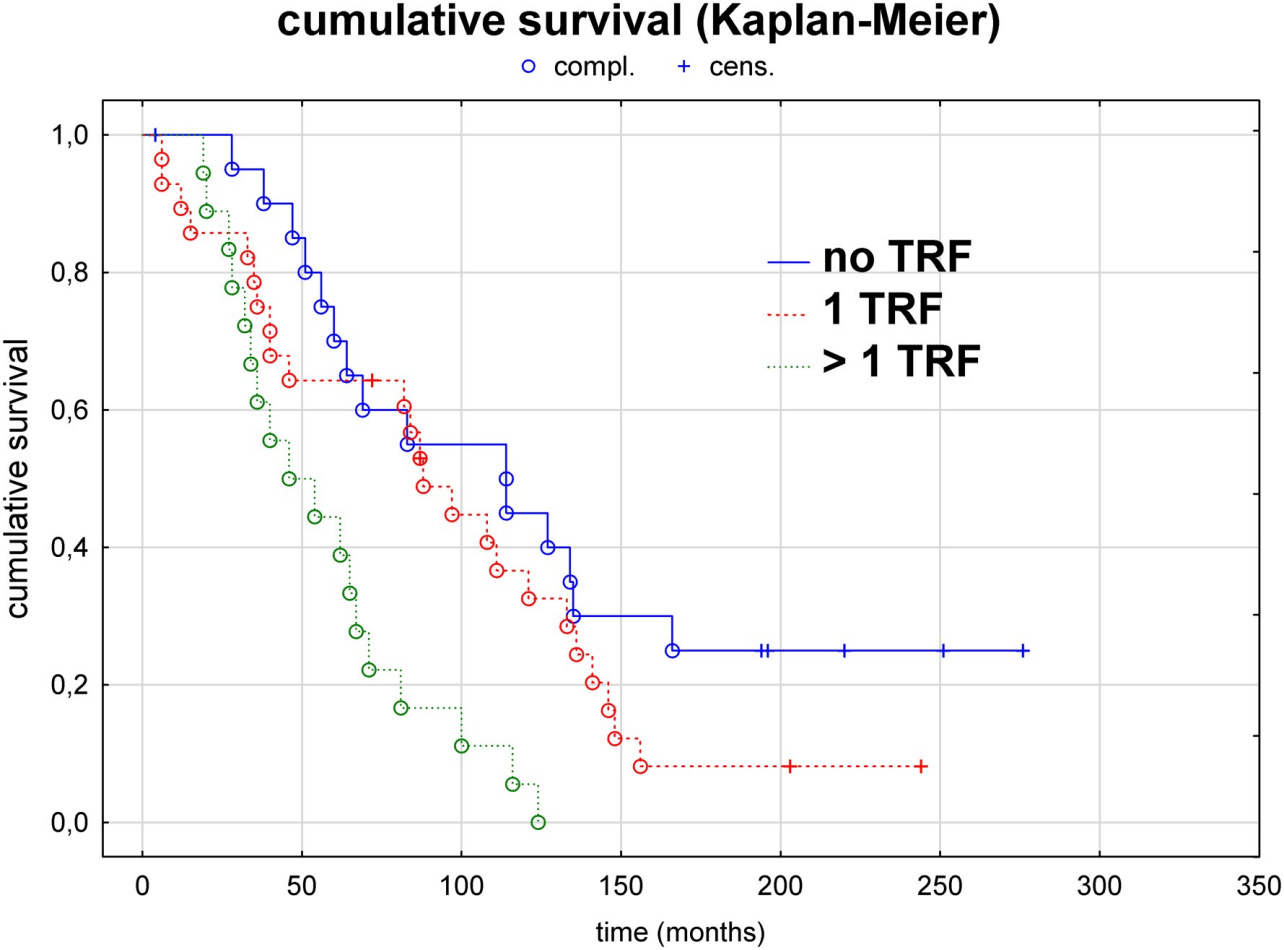
Survival analysis (Kaplan–Meier estimate and log-rank test) of patients with no thrombophilic risk factor (TRF) compared to patients with 1 TRF and patients with more than 1 TRF. Patient survival duration is significantly shorter in patients with more than 1 TRF than in patients without a TRF (*P*<0.01) or 1 TRF (*P*<0.02); however, the difference between the latter patients is not significant (*P* = 0.175). Survival duration is significantly longer in patients without a TRF than in patients with a TRF (*P*<0.02; not shown).

Conversely, Kaplan–Meier analyses with a log-rank test of patients without a TRF and those with TRFs showed a significantly shorter survival time in patients with a TRF (*P*<0.05). Additionally, survival was significantly shorter in patients with more than 1 TRF than in patients without a TRF (*P*<0.01) or those with 1 TRF (*P*<0.02), whereas there was no significant difference in survival between patients without a TRF and those with 1 TRF (*P* = 0.175) (Fig 4).

**Fig 5:**
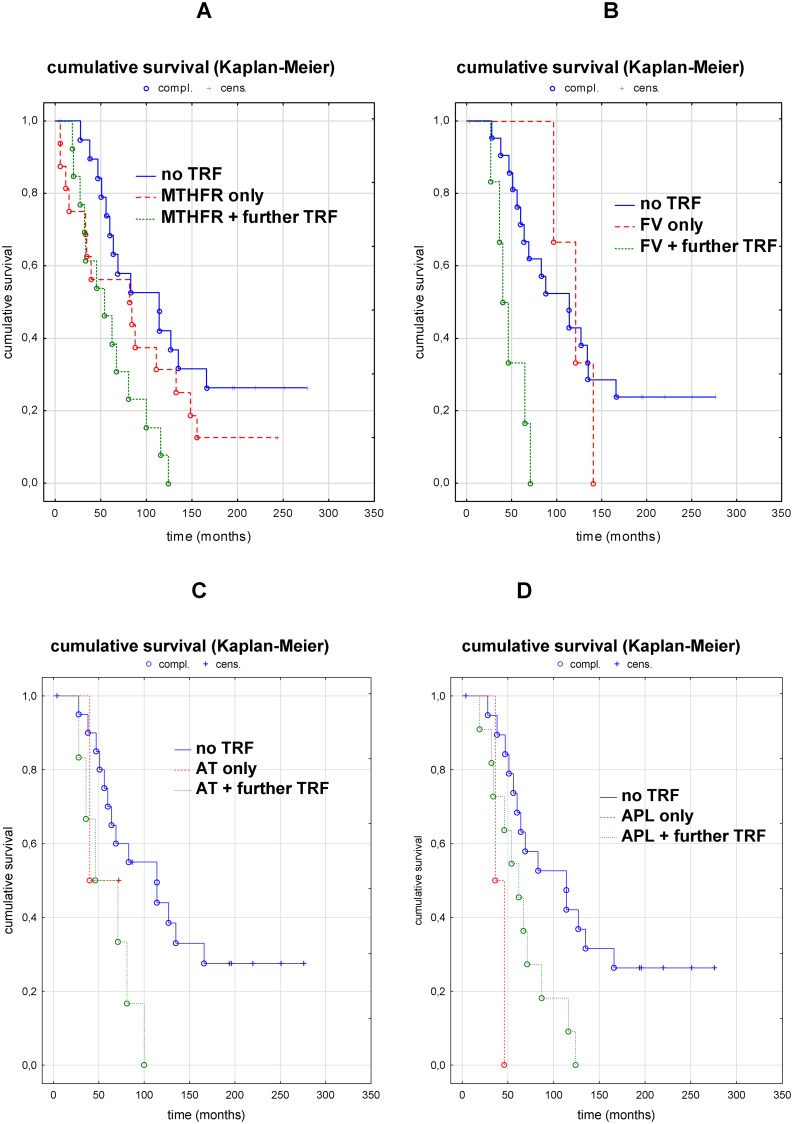
Survival analysis (Kaplan–Meier estimate and log-rank test) of patients for thrombophilic risk factors (TRFs) in detail. (A) MTHFR: Survival tended to be shorter in patients with only a *MTHFR* mutation (MTHFR only) than in patients without a TRF, but without statistical significance, whereas survival was significantly shorter in patients with a further coagulation disorder (MTHFR + further TRF) than in patients without a TRF (*P<*0.02). (B) Factor V (FV) mutation: Survival was not significantly different between patients with only a FV mutation (FV only) and patients without a TRF, whereas survival was significantly shorter in patients with a further coagulation disorder (FV + further TRF) than in patients without a TRF (*P<*0.05). (C) Antithrombin (AT) deficiency: Survival was not significantly different between patients with only an AT deficiency (AT only) and patients without a TRF, whereas survival tended to be shorter in patients with a further coagulation disorder (AT + further TRF) than in patients without a TRF (*P =* 0.054). (D) Antiphospholipid (APL) antibodies: Survival tended to be shorter in patients with only APL antibodies (APL only) than in patients without a TRF, but without statistical significance, whereas survival was significantly shorter in patients with a further coagulation disorder (APL + further TRF) than in patients without a TRF (*P<*0.05).

On evaluating the effects of various TRFs in detail, we did not observe a significant effect of any single TRF on patient survival. However, survival was significantly shorter in patients in whom a *MTHFR*-mutation, a factor V mutation, or APL-Abs occurred together with at least 1 further TRF than in patients with no TRF (log-rank test *P*<0.02, *P<*0.05, and *P*<0.05, respectively), while survival time tended to be shorter in patients with AT deficiency and a further TRF than in patients with no TRF (*P =* 0.054). Kaplan–Meier estimates of the TRFs with the highest prevalence are shown in Fig 5.

In summary, our results suggest that TRFs have a significant impact on survival time, particularly when more than 1 TRF is present, irrespective of the underlying entity.

## Discussion

Patients with endstage renal disease are in a substantial procoagulable state. Coagulatory activity is further stimulated by hemodialysis, accompanied for example by elevated levels of prothrombin fragments and thrombin anti-thrombin-complexes [25]. In this study, we focused on the role of various inherited and acquired TRFs in vascular access occlusion and patient survival.

Consistent with the results in other studies [4–7], the prevalence of TRFs was significantly higher among HD-patients in this study than among the general population [2, 22–24]. In particular, acquired or partially acquired TRFs were clearly enhanced (Table 1). The higher prevalence of AT, protein S, and protein C deficiencies in hemodialysis patients might be explained by increased inhibitor consumption owing to a state of increased coagulation activity resulting from chronic kidney disease (CKD) and dialysis. Ghisdal et al. [7] observed a higher prevalence of the aforementioned acquired TRFs in patients with stage V CKD than in the general population, and the prevalence was even higher in patients on hemodialysis or peritoneal dialysis. Moreover, Ghisdal et al. [7] found a significant drop in these TRFs almost to the values in the general population within 1 month after kidney transplantation. The relatively higher prevalence of inherited factor V Leiden and factor II mutations in this study when compared to that in other studies was presumably random, whereas the prevalence of *MTHFR* mutations was similar to that reported in the general population [13, 26, 27].

The primary shunt occlusion rate within the first 2 years of hemodialysis was well within the range reported by others [15–19]. In younger patients, we observed a higher shunt survival rate, which is consistent with the data reported by Rodriquez et al. [28].

Here, the presence of TRFs was a major risk factor for shunt patency. Only very few patients exhibited other thrombotic events, suggesting that these disorders may manifest in particular conditions, such as artificial AVFs. Our results are consistent with the findings in the study by Knoll et al. [6], where positive correlations were identified between the number of coagulation disorders and vascular access problems.

On examining the impact of a single TRF on shunt occlusion, we found a significant effect of the *MTHFR* C677T mutation, which is consistent with the result of the study by Fukasawa et al. [13]. However, in contrast to the findings of the study by Födinger et al. [29], we observed no significant difference in the homocystein level between patients with and those without shunt occlusion. In agreement with the findings of the study by Lee et al. [27], we detected no significant difference in the homocysteine level among the 3 *MTHFR* genotypes in patients with end-stage renal disease, which is considered to be relevant in the pathogenesis of *MTHFR*-related thrombotic events [30].

In patients with factor V Leiden, we observed a high shunt occlusion rate at a APC sensitivity ratio of 1.62, similar to the finding (ratio of 1.45) by Bremer and Schäfer [31], while Födinger et al. [11] did not find such an association; however, in this study, the ratio was 2.31.

Similar to the findings of the study by LeSar et al. [12], the presence of APL-Abs was a risk factor for arteriovenous access thrombosis. Valeri et al. [32] found no effect on AVF survival but noted shorter AV graft survival in patients with aCL-IgG.

In summary, we found that TRFs, irrespective of their entity, had a relevant impact on shunt survival.

As vascular access problems are associated with high morbidity [14], they might influence patient survival. An increased rate of AVF failure owing to TRFs could be of pathogenetic importance. In addition, TRFs are associated with high mortality in the general population [1–3]. To best of our knowledge, this is the first study to assess the effects of TRFs on HD-patient survival in a long-term follow-up and furthermore to assess whether early AVF occlusion is a significant risk factor for HD-patient survival. Although increased morbidity associated with vascular access problems has previously been suspected [14], we observed no significant effect of early shunt occlusion on patient survival. Conversely, we found significant effects of TRFs on patient survival, particularly with the presence of more than 1 TRF. The 2 surviving patients who were on hemodialysis after the observation period did not have coagulation disorders, just as 3 of the 5 patients with a kidney graft who were alive. The 2 surviving transplanted patients with TRFs showed *MTHFR* mutations.

The particularly high survival rate in patients with a kidney graft might be associated with the better overall survival of transplanted patients than dialysis patients [33]. An at least partial correction of acquired TRFs by renal transplantation, as reported by Ghisdal et al. [7], suggests that this phenomenon might also contribute to the improved survival of transplanted patients.

Our results suggest that TRFs have an important impact on the survival of HD-patients independent of early shunt failure. We could not find a significant effect of a single TRF on patient survival. Limitations of this study should be noted. The sample size of this study is moderate, similar to almost all studies in this field. This might explain why we could not find a significant effect of a single TRF on vascular access occlusion and patient survival. However, as outlined above, the prevalence of TRFs and comorbidities in this study were well within the range reported by other researchers for hemodialysis patients [4–6]. We refrained from showing dichotomized data of hypertension or smoking, as exposure to these factors can vary greatly from individual to individual. We therefore preferred to present the main consequences of these risk factors, coronary heart disease and stroke, which were as well in the range as reported by others [4–6]. Performing thrombophilia testing only once is also a limitation. Repeated testing for acquired hypercoagulabilty e.g. antiphosphospholipid antibodes at least after 12 weeks is recommended [34]. The proportion of HD-patients positive for antiphospholipid antibodies was also within the range reported by others for this patient group [10, 32, 35–38]. In summary, this suggests that we examined a quite representative group of HD-patients. It is also possible that acquired TRFs were elevated at the time of vascular access thrombosis but normalized at the time of measurement. If this was the case, it would further emphasize the relevance of these TRFs.

The observational design of this study is a further limitation. For example, it doesn’t adequately allow the evaluation of the role of antithrombotic agents. This would require a detailed specification of their application. We therefore refrained from listing them. So far, a positive effect of any antithrombotic therapy to increase the patency of AVF or transplants in the overall population of HD-patients has not been convincingly demonstrated [39–41].

The significant effects of TRFs observed in this population indicate that these factors are of clinical importance, although the reader should be aware of the wide CI. Thus, TRFs in HD-patients should be considered as important risk factors not only for shunt occlusion but also for shortened survival in these patients. To reduce these risks, this observational study could not provide an optimal strategy. For example, the role of antithrombotic therapies in this subgroup of patients should be clarified, as such an approach did not show any significant benefit in the total population of HD-patients, as previously described. Randomized trials are needed to definitively determine the indications and potential strategies of therapeutic interventions.

In conclusion, our results show that TRFs occur very frequently in HD-patients and have important effects on both early shunt occlusion and patient survival. Thus, TRFs have effects on morbidity and mortality in these patients, although early AVF-failure has no significant impact on patient survival. Our findings could provide a basis for the development of strategies to improve the negative effects of coagulation disorders in HD-patients.
